# Long-Term Amelioration Practices Reshape the Soil Microbiome in a Coastal Saline Soil and Alter the Richness and Vertical Distribution Differently Among Bacterial, Archaeal, and Fungal Communities

**DOI:** 10.3389/fmicb.2021.768203

**Published:** 2022-01-11

**Authors:** Ruibo Sun, Xiaogai Wang, Yinping Tian, Kai Guo, Xiaohui Feng, Hongyong Sun, Xiaojing Liu, Binbin Liu

**Affiliations:** ^1^Anhui Province Key Laboratory of Farmland Ecological Conservation and Pollution Prevention, Key Laboratory of JiangHuai Arable Land Resources Protection and Eco-Restoration, College of Resources and Environment, Anhui Agricultural University, Hefei, China; ^2^Key Laboratory of Agricultural Water Resources, Hebei Key Laboratory of Soil Ecology, Center for Agricultural Resources Research, Institute of Genetics and Developmental Biology, Chinese Academy of Sciences, Shijiazhuang, China; ^3^School of Life Sciences and Engineering, Handan University, Handan, China; ^4^Xiong’an Institute of Innovation, Chinese Academy of Sciences, Xiong’an New Area, China

**Keywords:** soil salinity, saline soil amelioration, microbial diversity, microbial community assembly, amplicon sequencing

## Abstract

Globally soil salinity is one of the most devastating environmental stresses affecting agricultural systems and causes huge economic losses each year. High soil salinity causes osmotic stress, nutritional imbalance and ion toxicity to plants and severely affects crop productivity in farming systems. Freezing saline water irrigation and plastic mulching techniques were successfully developed in our previous study to desalinize costal saline soil. Understanding how microbial communities respond during saline soil amelioration is crucial, given the key roles soil microbes play in ecosystem succession. In the present study, the community composition, diversity, assembly and potential ecological functions of archaea, bacteria and fungi in coastal saline soil under amelioration practices of freezing saline water irrigation, plastic mulching and the combination of freezing saline water irrigation and plastic mulching were assessed through high-throughput sequencing. These amelioration practices decreased archaeal and increased bacterial richness while leaving fungal richness little changed in the surface soil. Functional prediction revealed that the amelioration practices, especially winter irrigation with saline water and film mulched in spring, promoted a community harboring heterotrophic features. β-null deviation analysis illustrated that amelioration practices weakened the deterministic processes in structuring coastal saline soil microbial communities. These results advanced our understanding of the responses of the soil microbiome to amelioration practices and provided useful information for developing microbe-based remediation approaches in coastal saline soils.

## Introduction

High salt concentrations are one of the key factors limiting crop growth in coastal saline soil (Zhang et al., 2015). Although washing with fresh water is effective for reducing the salt concentration, a shortage of fresh water in coastal areas precludes the application of this technique (Li et al., 2008). Alternative methods using drip irrigation with saline water for salinity control have been developed (Sun et al., 2012; Li et al., 2017b); however, these systems are prone to suffer from emitter clogging (Yavuz et al., 2010), although some flushing regimes have been developed to cope with this issue (Feng et al., 2017; Zhangzhong et al., 2018). We have recently demonstrated that freezing saline water irrigation was an effective method for reducing salinity in surface saline soil, which has been illustrated both in a lab-scale column experiment (Li et al., 2008) and in field experiments (Guo and Liu, 2015, 2021). In combination with subsequent plastic mulching, which could help maintain the low soil salinity by preventing evaporation and salt backflow after infiltration, this technique increased the abundance of bacteria and fungi, enhanced soil respiration and facilitated seed germination in cotton (Guo and Liu, 2015; Li et al., 2017a).

The freezing saline water irrigation technique involves the use of saline water during winter to form an ice layer on the surface of the soil. As saline water freezes, the salt is not incorporated into the ice crystal but concentrated in the interstitial liquid brine (Notz and Worster, 2009). The resulting saline ice is therefore a mixture of freshwater ice crystals, brine pockets, air and solid impurities (Xie et al., 2009; Gu et al., 2012). Previous research has revealed that as the temperature gradually increases in the spring, the ice salinity declines with decreasing ice volume (Guo and Liu, 2015), possibly due to the brine pockets draining from the ice under the influence of gravity (Gu et al., 2012) and/or flushed out by relatively fresh surface meltwater (Notz and Worster, 2009). During this process, salinity gradually declined in the percolated meltwater, which infiltrated into the underlying saline soil and eventually resulted in a relatively low salinity in the surface soil to facilitate seed germination and crop growth. Although this method could dramatically reduce the salinity in surface soil, due to the shallow, high salinity groundwater table in coastal areas, saline soil treated solely with freezing saline water irrigation did not support plant (cotton) growth, as indicated in prior studies (Guo and Liu, 2015; Li et al., 2017a). To maintain the low salinity level achieved by infiltration of meltwater from saline ice, mulching (M) has been introduced and proven to be an efficient way to control the salinity in the root zone (Guo and Liu, 2015). Mulching could reduce soil-water evaporation and thereby prevent soluble salt from moving up with water flow and accumulating in the topsoil (Sadegh-Zadeh et al., 2009; Fall, 2017).

While salinity is altered by amelioration practices, other physical and chemical properties are also inevitably affected, as indicated in our previous studies (Guo and Liu, 2015; Li et al., 2017a). In addition to soil physicochemical characteristics, soil microbial properties have gained attention recently and have been increasingly used as bioindicators of soil quality (Epelde et al., 2014). Previous studies have demonstrated that changes in microbial community composition during saline soil amelioration are concomitant with changes in soil salinity or other soil properties (Elmajdoub and Marschner, 2015; Lu et al., 2015; Wang et al., 2020). However, how soil microbial community responds to freezing saline water irrigation and mulching in coastal saline areas remains unclear. In particular, due to alterations in salt distribution and soil aggregation along the soil profile during these amelioration processes (Ju et al., 2018), it is of great interest to understand the accompanying vertical changes in soil microbial properties (Zahran, 1997).

Soil microbes play important roles in driving soil biogeochemical cycling (Nielsen et al., 2011; Graham et al., 2014) and maintaining soil quality (Kennedy and Smith, 1995; Clergue et al., 2005). In agricultural systems, it has been well documented that soil microbial communities are sensitive to a number of environmental factors, including soil pH (Valentin-Vargas et al., 2014; Sun et al., 2015b), moisture (Evans and Wallenstein, 2012; Ma et al., 2015), and temperature (Lauber et al., 2013; Auffret et al., 2016), and could be altered greatly by agricultural practices, such as fertilization (Poulsen et al., 2013; Hartmann et al., 2014; Sun et al., 2015b,^2016^) and tillage (Zuber and Villamil, 2016; Sun et al., 2018). Salinity is the major stress for microorganisms in saline soils and has been found to be negatively associated with microbial abundances and activities (Siddikee et al., 2011; Wu et al., 2015; Singh, 2016). As reviewed by Singh (2016), prior studies have demonstrated that bacterial growth clearly responded to salt concentration, but soil salinity was not proposed as a decisive factor for alterations in bacterial growth or community structure in the investigated soils because microbial communities have evolved the ability to survive saline conditions through physiological and structural modifications (Zahran, 1997). These survival strategies increased the uncertainty as to how soil microbial populations respond to soil salinity changes. In particular, how soil microbial communities shift and reassemble with decreasing salinity achieved by long-term saline soil amelioration remains poorly understood.

Since coastal areas are located close to the sea, a considerable portion of microorganisms in coastal soils are marine-sourced, and we hypothesized that the effects of marine environments on the microbial community in coastal saline soil will be weakened and microorganisms living in lower-salinity environments will be enriched. To test this hypothesis and bridge the knowledge gap regarding the responses of soil microorganisms to saline soil amelioration, microbial community composition, diversity and assembly mechanisms under different amelioration practices were evaluated through high-throughput sequencing and bioinformatic analyses.

## Materials and Methods

### Experimental Design and Soil Sampling

Details of the study site and experimental design were described in previous studies (Guo and Liu, 2015; Li et al., 2017a). In brief, the experiment was set up at a coastal saline land station (Haixing station) of the Chinese Academy of Sciences on the coastal saline plain of the Bohai Sea (117°33′, 38°10′N). Agronomic practices, weather conditions and soil water contents have been described in our previous study (Guo and Liu, 2015; Li et al., 2017a); the mean annual temperature in this area is 12.1°C and average annual precipitation is 582.3 mm. The soil in this area is a chloride-type saline soil with a salinity of 7.74 ± 1.83 (g/kg, total concentration of all dissolved salts) (Li et al., 2017a), in which sodium and chloride accounted for more than 75% of the total ions (Guo and Liu, 2015). The experiment was initiated in 2008 and included four treatments with three replicates (plots): (1) Control (wasteland without amelioration); (2) winter irrigation with saline water (salinity of 9.59 g L^–1^; WI); (3) film mulched (non-degradable plastic film, 0.07 mm) in spring (M); and (4) winter irrigation with saline water and film mulched in spring (WIM). Saline water was irrigated in winter when the temperature was below –10.3°C to form a 180 mm ice layer. Plastic mulching was applied in the following year after the ice thawed, and the meltwater infiltrated into the soil (early March).

Soil samples from each plot were collected from three layers (0–10, 10–20, and 20-30 cm) after cotton harvest in October 2016. Seven soil cores along a zigzag line were collected from each plot by auger boring and completely homogenized as a single sample. Foreign matter, such as stones and plant residues, was removed by sieving through 2-mm meshes. To avoid cross-contamination, the auger and meshes were scrubbed with 75% alcohol, rinsed with sterile water, and then dried completely before first use and between different soil samples (Sun et al., 2015b). One portion of each soil sample was stored at 4°C for biogeochemical measurement, while the other portion was stored at −80°C for molecular analysis.

### Soil Property Measurements and DNA Extraction

Soil properties were evaluated as described previously (Chu and Grogan, 2010; Sun et al., 2015b). Soil pH and electrical conductivity (EC) were measured using a pH meter (FE28; Mettler-Toledo, Zurich, Switzerland) and a conductivity meter (DDS-307A; INESA Scientific Instrument Co., Ltd, Beijing, China), respectively, at a soil:water ratio of 1:5 (dry weight/volume). Soil total carbon (TC) and nitrogen (TN) were measured using a CHNOS elemental analyzer (Vario MAX; Elementar, Langenselbold, Germany). The dichromate oxidation method was used to measure the soil organic carbon (SOC) content (Shaw, 1959). Total DNA was extracted from 0.5 g of fresh soil using a FastDNA SPIN Kit (MP Biomedicals, Santa Ana, CA, United States) following the manufacturer’s instructions.

### Molecular Analyses

The modified primers 515f/806r (5′-GTGYCAGCMGCCGC GGTAA-3′/5′-GGACTACNVGGGTWTCTAAT-3′), targeting the V4 region of the 16S rRNA gene, and ITS1f/ITS2 (5′-CTTGGTCATTTAGAGGAAGTAA-3′/5′-GCTGCGTTCTTCA TCGATGC-3′), targeting the internal transcribed spacer (ITS) of fungal rRNA, were used for analyses of the prokaryotic (archaeal and bacterial) and fungal communities, respectively (Walters et al., 2016). Polymerase chain reaction (PCR) and sequencing protocols were described in a previous study (Sun et al., 2018). Briefly, each 25 μl PCR mixture contained 12.5 μl of PCR premix (Ex Taq™; Takara, Shiga, Japan), 0.5 μl (10 μM) of the forward and reverse primers (final concentration: 0.2 pmol ⋅ μl^–1^ each), 0.5 μl of DNA template (20 ng), and 11 μl of sterile double distilled water. The following thermocycling conditions were used: initial denaturation at 94°C for 10 min, followed by 30 amplification cycles of denaturation at 94°C for 30 s, annealing at 50°C/56°C (16S rRNA/ITS) for 45 s, extension at 72°C for 1 min, and a final extension at 72°C for 10 min. High-throughput sequencing was performed using the Illumina HiSeq 2000 platform (Illumina, San Diego, CA, United States). The sequencing data obtained in this study have been deposited in the European Nucleotide Archive (accession No. PRJEB29290).

### Bioinformatics Analyses

Bioinformatics analyses were performed using Quantitative Insights Into Microbial Ecology (QIIME, v 1.9.1) (Caporaso et al., 2010) according to previously described methods (Sun et al., 2018). Briefly, after removing the adaptor and barcode bases using cutadapt (Martin, 2011), the paired-end reads were joined using the fastq-join method. Low-quality sequences (Phred quality score < 20, length < 200 bp) and chimeras were filtered out. The clean sequences were subsequently clustered into operational taxonomic units (OTUs) based on a similarity threshold of 0.97 using the UCLUST method (Edgar, 2010). The taxon of each OTU was assigned using RDP Classifier (Wang et al., 2007) based on a representative sequence of the OTU (the most abundant sequence within the OTU). Singletons and OTUs that failed to be assigned to Archaea, Bacteria, or Fungi at the kingdom level were removed. The OTU tables of the archaeal, bacterial, and fungal communities were subsampled to 1,200, 23,000, and 12,000 sequences per sample, respectively, for statistical analyses. OTU richness and Heip’s evenness index (Heip, 1974) were calculated to compare the α diversities of the microbial communities in different treatments.

### Prediction of Functional Profiles

The functional profiles of the prokaryotic communities were annotated using Functional Annotation of Prokaryotic Taxa (FAPROTAX) (Louca et al., 2016), while the ecological guilds of the fungal communities were assigned using FUNGuild (Nguyen et al., 2016). Fungal OTUs with an assigned confidence ranking of “possible” from FUNGuild were considered to be unassigned and excluded from downstream analysis (Janoušková et al., 2018). These software platforms provided useful approaches for assessing microbial community function using phylogenetic marker genes. However, the potential differences in phylogenetic trait conservatism (Martiny et al., 2013, 2015; Louca et al., 2018), the limited precision in taxonomic annotation using phylogenetic marker genes and the limited coverage of the database (Nguyen et al., 2016) could strongly impact the reliability of the functional prediction. Quantitative PCR (qPCR) targeting a functional gene (*amoA*, which encodes ammonia monooxygenase responsible for catalyzing ammonia oxidization) was therefore conducted using a previously described protocol (Sun et al., 2015a) to confirm the results of functional prediction.

### Statistical Analyses

Statistical analyses and generation of the figures were performed in R (v 3.4.3) using the relevant packages. Significant differences between treatments for the assayed variables, such as soil properties and microbial diversity indices, were evaluated using the Kruskal–Wallis rank sum test (Corder and Foreman, 2009) with the “dplyr” package. Heatmaps showing the composition of the microbial communities were drawn using the “pheatmap” package, and the treatments were clustered using the unweighted pair group method with arithmetic mean (UPGMA) (Milligan, 1985). Non-metric multidimensional scaling (NMDS) (Clarke, 1993) based on Bray–Curtis distance was performed using the “vegan” package. Analysis of similarity (ANOSIM) was used to test the significance of dissimilarity (measured by Bray–Curtis distance) in microbial communities among treatments and soil depth with 999 permutations. The log response ratio (LRR) (Hedges et al., 1999) showing the effect size of the amelioration treatment on soil microbial taxa was calculated with bias correction using the “ARPobservation” package. Spearman’s correlation coefficients between soil microbial communities and soil properties were assessed by Mantel tests using the “vegan” package, and the best subset of environmental variables maximally correlating with community dissimilarities was determined using the bioenv function. The roles of deterministic and stochastic processes in soil microbial community assembly were assessed using the β-null deviation method according to Tucker et al. (2016). Bray–Curtis dissimilarity was calculated to quantify the total changes in the soil microbial communities between different treatments.

## Results

### Soil Properties Under Different Amelioration Regimes

The observed changes in soil properties were summarized in Table 1. Soil electrical conductivity (EC) in the 0–10 cm layer was significantly lower in the ameliorated soils than in the Control, suggesting that the amelioration practices successfully reduced the salt concentration in the surface soil. The plant (cotton) successfully grew in the M and WIM treatments, but not in the Control or WI treatments. Soil moisture (SM) varied little among treatments and soil layers, and SM in the treatments including mulching (M and WIM) was higher than that of WI, indicating that mulching was effective in the maintenance of soil moisture. The surface soils in treatments with mulching showed significantly higher total carbon (TC) (0–10 cm) than that in the Control, and no significant changes in TC between WI and the Control were observed. This may have been due to the growth of cotton in the M and WIM treatments (Guo and Liu, 2015).

**TABLE 1:** Soil properties in different amelioration treatments.

Factor	Soil layer (cm)	Control	WI	M	WIM
SM (%)	0–10	23.53 ± 0.66^ab†^	22.8 ± 0.47^b^	23.36 ± 0.57^ab^	24.25 ± 0.62^a^
	10–20	23.59 ± 0.05^a^	22.82 ± 1.04^a^	23.41 ± 0.29^a^	23.84 ± 0.45^a^
	20–30	22.78 ± 0.59^b^	21.89 ± 0.75^b^	23.73 ± 0.46^a^	23.77 ± 0.69^a^
pH	0–10	8.41 ± 0.14^b^	8.77 ± 0.04^a^	8.34 ± 0.25^b^	8.41 ± 0.10^b^
	10–20	8.92 ± 0.12^a^	9.11 ± 0.12^a^	9.13 ± 0.14^a^	9.03 ± 0.15^a^
	20–30	9.02 ± 0.26^a^	9.10 ± 0.11^a^	8.88 ± 0.12^a^	9.09 ± 0.12^a^
EC (μS ⋅ m^–1^)	0–10	4450 ± 620^a^	3115 ± 85^b^	2173 ± 481c	1307 ± 159^d^
	10–20	1354 ± 391^a^	1047 ± 149^a^	1207 ± 59^a^	1161 ± 119^a^
	20–30	1039 ± 331^a^	1124 ± 5^a^	1259 ± 77^a^	1118 ± 31^a^
TC (%)	0–10	1.54 ± 0.03^b^	1.52 ± 0.06_b_	1.73 ± 0.04^a^	1.63 ± 0.08^a^
	10–20	1.51 ± 0.15^a^	1.64 ± 0.01^a^	1.54 ± 0.10^a^	1.51 ± 0.08^a^
	20–30	1.35 ± 0.13^a^	1.33 ± 0.10^a^	1.40 ± 0.12^a^	1.41 ± 0.13^a^
TN (%)	0–10	0.091 ± 0.001^a^	0.086 ± 0.007^a^	0.091 ± 0.002^a^	0.094 ± 0.010^a^
	10–20	0.068 ± 0.005^a^	0.062 ± 0.009^ab^	0.072 ± 0.003^a^	0.063 ± 0.003^b^
	20–30	0.072 ± 0.010^a^	0.077 ± 0.009^a^	0.069 ± 0.004^a^	0.073 ± 0.008^a^
SOC (%)	0–10	0.654 ± 0.045^b^	0.617 ± 0.051^b^	0.788 ± 0.019^a^	0.646 ± 0.032^b^
	10–20	0.563 ± 0.013^b^	0.710 ± 0.131^a^	0.472 ± 0.011^c^	0.566 ± 0.068^b^
	20–30	0.569 ± 0.032^a^	0.563 ± 0.051^a^	0.528 ± 0.051^a^	0.526 ± 0.032^a^

*^†^Means within a row sharing a letter in common are not significantly different from each other according to the Kruskal-Wallis rank sum test (P > 0.05).*

*WI, freezing saline water irrigation; M, plastic mulching; WIM, WI+M. SM, soil moisture; EC, electrical conductivity; TC, total carbon; TN, total nitrogen; SOC, soil organic carbon.*

### Effect of Amelioration on Soil Microbial Composition and α Diversity

**FIGURE 1:**
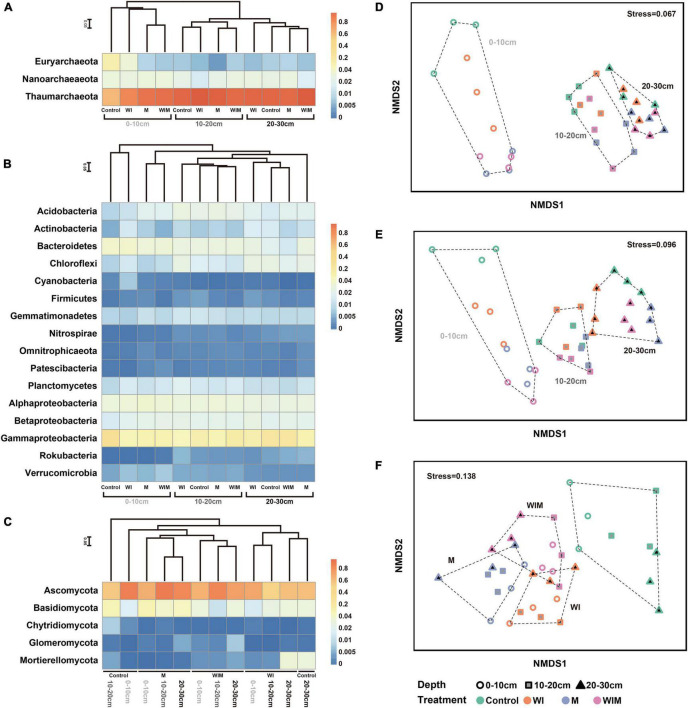
Heatmap showing the dominant phyla of soil archaea **(A)**, bacteria (class for Proteobacteria) **(B)** and fungi **(C)** under different treatments. Non-metric multidimensional scaling (NMDS) ordination of soil archaeal **(D)**, bacterial **(E)**, and fungal **(F)** communities. Both the cluster dendrogram and NMDS were generated based on Bray–Curtis distance. WI, freezing saline water irrigation; M, plastic mulching; WIM, WI + M.

After sequencing and quality filtering, 396,660 16S rRNA gene sequences were retained. Based on taxonomic annotation results, the archaeal and bacterial communities were split and analyzed separately. The soil archaeal community was dominated by Thaumarchaeota, Nanoarchaeota, and Euryarchaeota, which accounted for 84.54, 8.54, and 5.95% of the total archaeal reads, respectively (Figure 1A). The most abundant phylum in the bacterial community was Proteobacteria, accounting for 47.84% of the total bacterial reads, followed by Bacteroidetes, Acidobacteria, Chloroflexi, Planctomycetes, Gemmatimonadetes, and Actinobacteria, accounting for 10.35, 6.29, 5.81, 4.86, 4.18, and 3.76%, respectively. These seven phyla accounted for more than 81% of the total bacterial community in relative abundance (Figure 1B).

For the fungal community, 817,019 high-quality ITS sequences were analyzed, and the majority were assigned to the phylum Ascomycota, accounting for 64.14% of the total reads. Basidiomycota was the next most dominant fungal phylum and accounted for 12.74% of the total reads, and 6.22, 2.83, and 0.75% of the reads were assigned to the phyla Mortierellomycota, Glomeromycota, and Chytridiomycota, respectively.

Non-metric multidimensional scaling ordination based on Bray–Curtis distance was performed to assess the response patterns of the soil microbial communities to different amelioration regimes (Figures 1D–F). The archaeal, bacterial, and fungal communities exhibited different patterns in the NMDS plot, and the soil archaeal and bacterial communities separated along the first axis, corresponding to soil depth from shallow to deep (Figures 1D,E). The separation of the archaeal and bacterial communities under different treatments was only observed in the surface soil (0–10 cm). These results indicated that soil prokaryotic communities greatly varied according to soil depth and that the amelioration practices impacted prokaryotic communities in the surface soil but had little effect on those in the subsoil. This was supported by the results of ANOSIM (analysis of similarities), which showed significant differences in the archaeal and bacterial communities among soil layers, whereas significant differences according to treatment regime were only observed in the surface soil (Supplementary Table S1). Unlike prokaryotic communities, soil fungal communities showed different patterns both across soil depths and among amelioration treatments (Figure 1F). The fungal communities clustered according to amelioration treatments and did not show clear association with any particular soil depth, indicating consistent effects of amelioration practices on fungal communities throughout the investigated soil layers. Additionally, ANOSIM revealed significant differences in the fungal communities among treatments in the same layer but no significant difference between soil layers receiving the same treatment (Supplementary Table S1).

**FIGURE 2:**
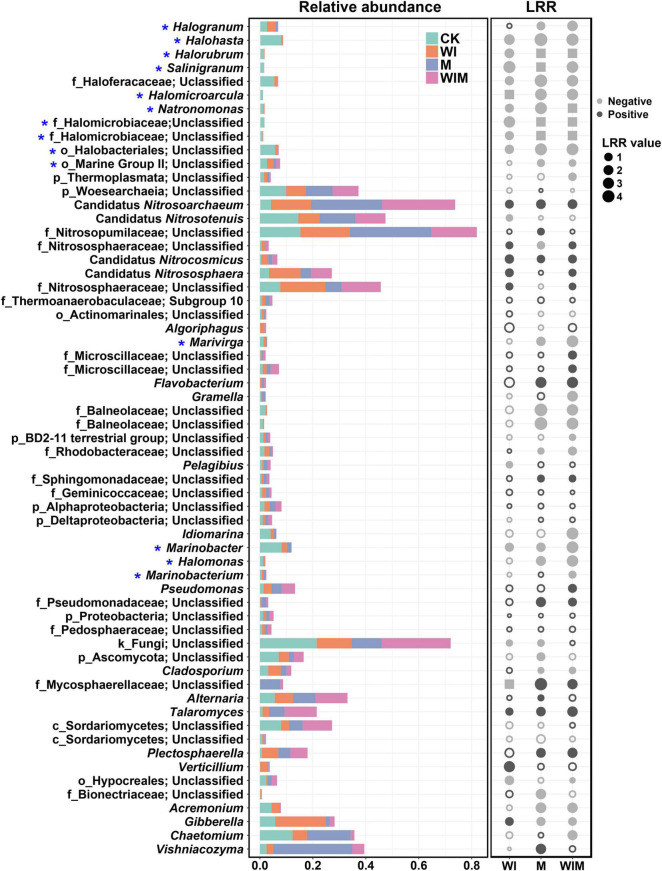
Comparisons of the dominant genera of the soil microbial community in the 0–10 cm layer for different treatments (stacked plot) and the log response ratio (LRR) (dot plot) between the ameliorated soils (WI, M, and WIM) and the Control soil. Taxonomic level: k, kingdom; p, phylum; c, class; o, order; and f, family. The gray color in the dot plot indicates negative values, and the black color indicates positive values. Solid dots indicate significant differences in the relative abundance compared with the Control soil according to the Kruskal–Wallis rank sum test (*P* < 0.05). The squares indicate that the genus was not detected (relative abundance: 0) in the amended soils. * Halophilic or haloduric taxa. WI, freezing saline water irrigation; M, plastic mulching; WIM, WI + M.

To illustrate the responses of soil microbial taxa to the amelioration practices, changes in the relative abundances of the primary genera (≥1% in relative abundance) in the surface soil (0–10 cm) and their LRR relative to the Control were calculated (Figure 2). The distribution of genera of the archaeal, bacterial and fungal communities from the Control and three amelioration treatments was illustrated in Venn diagrams (Supplementary Figure S1). A number of the genera within the class Halobacteria, including *Halohasta*, *Halorubrum*, *Salinigranum* and several unclassified genera, declined in relative abundance in the three amelioration treatments compared with the Control treatment. In contrast, most of the genera within the class Nitrososphaeria, such as Candidatus *Nitrosoarchaeum*, Candidatus *Nitrocosmicus*, and Candidatus *Nitrososphaera*, were enriched. In total, the abundances of 40, 35, and 22 dominant genera were significantly altered by the WIM, M, and WI treatments, respectively, suggesting a stronger impact of the treatments with mulching (WIM and M) than that of the treatment without mulching (WI). This was supported by results showing that the Bray–Curtis distance of soil microbial communities between the treatments with mulching and the Control was greater than that between WI and the Control (Supplementary Table S2).

**FIGURE 3:**
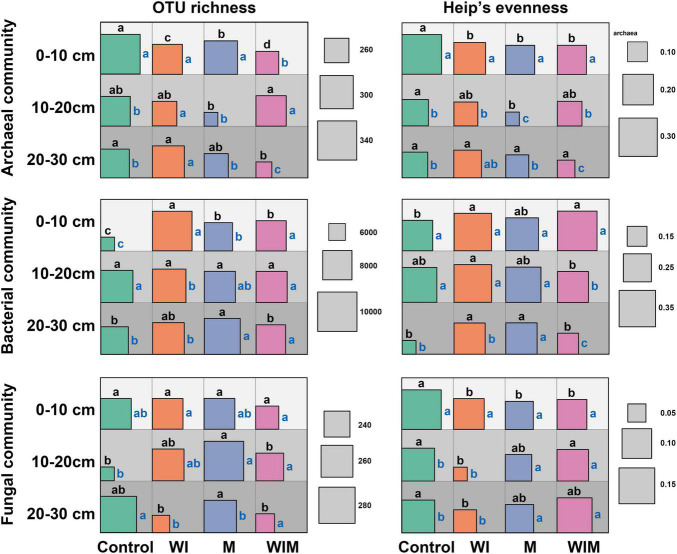
Diversity (OTU richness and Heip’s evenness) of soil archaeal, bacterial and fungal communities. Different letters on the top of the square indicate significant differences among treatments within the same soil layer, and those on the right side indicate significant differences among layers within the same treatment. The significance of the difference was tested according to the Kruskal-Wallis rank sum test (*P* > 0.05). WI, freezing saline water irrigation; M, plastic mulching; WIM, WI + M.

OTU richness and Heip’s evenness were calculated to evaluate changes in microbial α diversity under different amelioration treatments (Figure 3). Stronger impacts of amelioration treatments on soil archaeal and bacterial diversities were observed in the surface soil (0–10 cm) than in the subsoil layers. In the surface layer, the richness of the archaeal communities was significantly lower in the amelioration treatments than in the Control treatment. The reverse trend was discovered in the bacterial communities, and no significant changes were observed for the fungal communities. The evenness of archaeal and fungal communities was lower in the three amelioration treatments than in the Control.

### Microbial Community Assembly Under Different Amelioration Regimes

**FIGURE 4:**
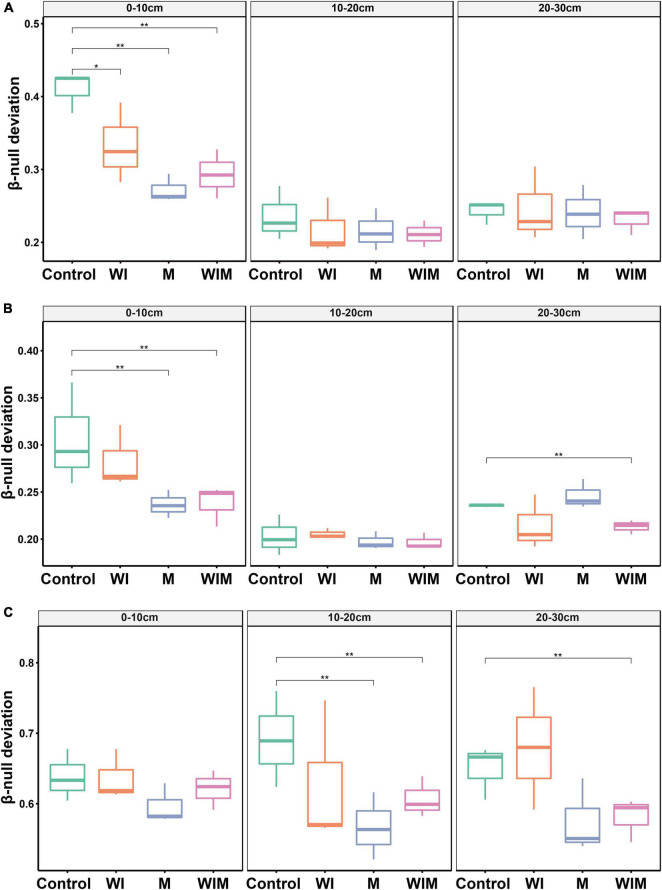
Abundance-based β-null deviation for archaeal **(A)**, bacterial **(B)**, and fungal **(C)** communities based on Bray–Curtis distance. * Indicates a significant difference between treatments as determined by Kruskal–Wallis rank sum test (*P < 0.05; **P < 0.01). WI, freezing saline water irrigation; M, plastic mulching; WIM, WI + M.

An abundance-based β-null deviation method was used to assess the contribution of stochastic and deterministic processes to the microbial community assembly. The β-null deviation values for all archaeal, bacterial and fungal communities were positive, suggesting that microbial composition was dissimilar in the randomly assembled community produced by the null model (Figure 4). In addition, the β-null deviation values varied among treatments, and compared to the Control soils, a general declining trend was observed in the amelioration treatments and suggested a weaker influence of deterministic assembly processes on microbial communities in the ameliorated soils compared with the Control.

### Functional Prediction of Prokaryotic and Fungal Communities

**FIGURE 5:**
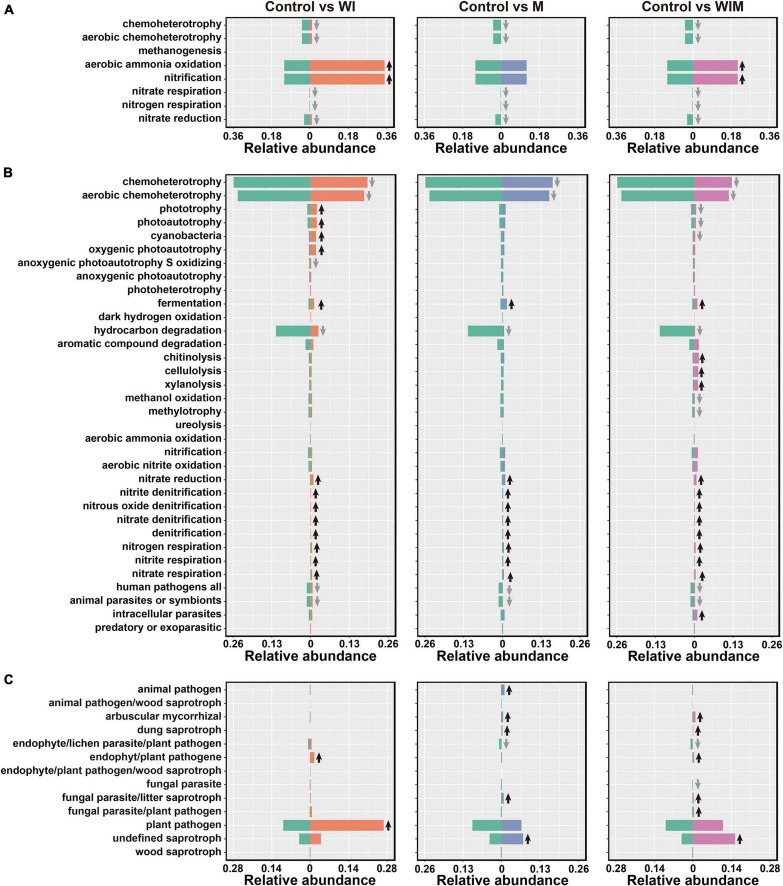
Comparisons of functional community profiles of archaea **(A)**, bacteria **(B)**, and fungi **(C)** for different treatments using FAPROTAX (for the archaeal and bacterial communities) and FUNGuild (for the fungal community). Black up and gray down arrows indicate significantly higher and lower relative abundances compared with the Control, respectively (*P* < 0.05, determined by Kruskal–Wallis rank sum test). WI, freezing saline water irrigation; M, plastic mulching; WIM, WI + M.

Using FAPROTAX and FUNGuild, the functions of prokaryotic and fungal taxa were predicted to reveal the changes in the function of the soil microbial communities. Eleven categories were predicted in the archaeal community, including eight categories detected in the 0–10 cm soil layer. The dominant functional group in the archaeal community was aerobic ammonia oxidation. This functional group was significantly elevated in both the WI and WIM treatments, as the corresponding taxon Nitrososphaeria dominated the respective archaeal communities (Figure 2 and Supplementary Table S3). However, decreases in other functions involved in nitrogen cycling, such as nitrate/nitrogen respiration and nitrate reduction and chemoheterotrophy, including aerobic chemoheterotrophy, were observed in response to all three amelioration practices (Figure 5A). For the bacterial community, the dominant functional group chemoheterotrophy decreased in the ameliorated soils, whereas most of the functions involved in nitrogen cycling were increased in the ameliorated soils (Figure 5B). Moreover, plant pathogens were the dominant guild with the highest relative abundance in the Control soil and showed a significant increase in the WI-treated soil. Additionally, many saprotrophs were increased in the M- and WIM-treated soils (Figure 5C).

### Correlations Between Soil Microbial Communities and Soil Properties

The 0–10 cm soil layer was chosen for correlation analyses because the greatest changes in the soil microbial communities and soil properties were detected in this layer (Figure 1 and Table 1). Both archaeal and bacterial communities were significantly correlated with a single soil factor, EC, with the best subset of soil variables having maximal correlation with archaeal and bacterial community dissimilarities containing only this single factor. In addition to EC, the fungal community showed a significant correlation with TN, with EC and TN as the best subset and showing the highest correlation with fungal community (Table 2). Analogously, the β-null deviation of the archaeal and bacterial communities was significantly correlated with EC, more than any of the other soil factors measured, however, no significant correlation was detected between the β-null deviation of the fungal community and any assayed soil properties (Table 2).

**TABLE 2:** Spearman’s correlation between soil microbial community structure (CS), β-null deviation (βD), and soil properties (0–10 cm soil layer).

Factor	Archaea		Bacteria		Fungi	
	CS	βD	CS	βD	CS	βD
SM	–0.074	0.063	–0.069	–0.441	–0.050	–0.294
pH	–0.171	0.166	–0.102	0.258	–0.196	–0.321
EC	**0.730**	**0.587**	**0.717**	**0.671**	**0.403**	0.217
TC	–0.130	–0.235	–0.053	–0.301	0.104	0.088
TN	0.133	–0.490	0.179	–0.336	**0.353**	–0.448
SOC	–0.066	–0.259	–0.055	–0.224	–0.174	–0.490
Best subset	EC *r* = 0.730		EC *r* = 0.717		EC, TN *r* = 0.486	

*Bold values indicate significant correlations (P < 0.05).*

*Correlation between microbial community structure and soil properties was performed by the Mantel test based on Bray–Curtis distance, and the best subset was determined using the bioenv model.*

*SM, soil moisture; EC, electrical conductivity; TC, total carbon; TN, total nitrogen; SOC, soil organic carbon.*

## Discussion

Saline soil remediation has recently gained increasing attention due to its potential for agricultural production (Li et al., 2017a), and a number of remediation techniques, including organic amendment (Wang et al., 2014; Zhang et al., 2016; Wu et al., 2018; Yang et al., 2018; Sun et al., 2020), phytoremediation (Hasanuzzaman et al., 2014; Shao et al., 2019; Wang et al., 2020), and microorganism inoculation (Zhang et al., 2014; Arora et al., 2016), have been developed recently. Freezing saline water irrigation, which utilizes local saline water, is an economical technique for saline soil remediation and has been successfully applied in saline soils of coastal areas (Guo and Liu, 2014) and inland irrigation areas (Guo and Liu, 2021). While the effects of this technique on the chemical and physical properties have been intensively investigated (Guo and Liu, 2015, 2021; Li et al., 2017a), sparse information is available about how the microbes in soil respond to amelioration practices. The current study filled this knowledge gap and, for the first time, illustrated different response patterns among bacteria, fungi and archaea.

In this study, the different responses of soil archaeal, bacterial and fungal communities to three amelioration practices were observed. The differences in the response patterns among the taxonomic groups have also been revealed in a previous study where saline soil was amended with biochar poultry-manure compost in conjunction with a pyroligneous solution (Lu et al., 2015). These differences might be associated with their community composition and different tolerances to salinity. The archaeal communities include many neutrophilic/halophilic taxa, such as *Halogranum*, *Halohasta*, *Halorubrum*, and *Salinigranum* (Figure 2), that can only survive in environments with a high salt concentration (Lizama et al., 2002; Kim et al., 2011; Mou et al., 2012; Corral et al., 2016; Wang et al., 2016). These taxa were absent in the ameliorated soil, where a much lower salt concentration than that of the original soil was achieved (Table 1), and resulted in a lower diversity in the archaeal community. The significantly positive correlation between archaeal richness and soil EC (Spearman’s correlation coefficient: 0.734, *P* < 0.05) also suggested that amelioration practices decreased archaeal diversity primarily by reducing soil salinity. Consistently, our recent study on phytoremediation of coastal saline soil has also demonstrated a significant decline in archaeal richness through planting salt-tolerant plants (Wang et al., 2020). However, the amelioration practices only slightly affected fungal richness (Figure 3). One possible reason for this result is that soil fungi are more resistant to salinity stress than soil bacteria (Rath et al., 2016), as fungi have a chitinous cell wall and different energy generation systems from bacteria, both of which offer protection against osmotic stress (Thornber, 1987; Strickland and Rousk, 2010; Rath et al., 2016). Another possible reason for this phenomenon is that halophilic taxa normally fluctuate dramatically along with salinity changes; however, only one known halophilic taxon, *Hortaea werneckii* (Plemenitaš et al., 2014), was detected in the soil fungal community. Therefore, changes in halophilic fungi, specifically, fungal species replacement in general, contributed minimally to the changes in community richness, and the shift in fungal communities between treatments primarily resulted from changes in species abundance. For bacterial communities, although the relative abundances of some halophiles, such as *Halomonas* (Gunde-Cimerman et al., 2018), also decreased in the ameliorated soil, the increased bacterial richness (Figure 3) associated with the amelioration treatments indicated that these practices promoted the colonization of new bacterial taxa. An increase in bacterial diversity was also discovered in saline soil remediation with farm manure (Shi et al., 2019) and with planting *Jerusalem artichokes* (Shao et al., 2019).

Understanding the mechanisms that shape soil microbial community structure is a central topic in microbial ecology (Zhou and Ning, 2017). The β-null deviation results showed that microbial communities in the studied saline soil deviated from the null model in which assembly occurred at random (Figure 4), indicating the profound contribution of the deterministic process governing microbial community assembly. This result was expected since the high concentration of salt is a strong filter for most microbial taxa. This notion was supported by the significant correlation between soil EC and the soil microbial community structure (Table 2). Accordingly, the decline in the importance of deterministic processes for community assembly in the ameliorated soils (Figure 4) could probably be due to the decrease in the salt concentration, which weakened niche selection and led to an environment suitable for more diverse microorganisms. Additionally, in the current study, plants (cotton) managed to grow in the treatments with mulching (M and WIM), and the nutrient input (especially carbon) from plants might have also contributed to the decrease in the importance of deterministic processes in microbial community assembly, as nutrient input has been perceived to enhance random changes in the relative abundances of taxa within a microbial community, weaken niche selection by reducing resource competition, and strengthen priority effects (Chase, 2007), thereby increasing the unpredictability of microbial composition and the stochasticity of microbial assembly (Zhou et al., 2014).

A number of species found in the Control soil have been found in sea water or marine sediment, such as *Marivirga* (Lin et al., 2015), *Gramella* (Lau et al., 2005), *Pelagibius* (Choi et al., 2009), *Idiomarina* (Ivanova et al., 2000), *Marinobacter* (Raddadi et al., 2017), and *Marinobacterium* (Chang et al., 2007), indicating the pronounced influence of the ocean on the microbial composition of coastal saline soil. However, the relative abundances of these taxa decreased in the ameliorated soils, especially in WIM-treated soil (Figure 2), suggesting that the amelioration practices weakened the effect of seawater on the microbial communities. A similar propensity was also observed in the functional prediction analyses, where the relative abundance of aerobic chemoheterotrophy, a function that has been found to be dominant in marine systems (Louca et al., 2016), was significantly decreased by amelioration (Figure 5).

Among the three amelioration treatments, WIM resulted in the greatest changes in soil microbial communities, with a decreasing trend in autotrophs and an increasing trend in heterotrophs in the community. This could be attributed to the plant (cotton) growth in WIM introducing exogenous carbon inputs that support the growth of heterotrophic microorganisms. Profound effects of plant growth on the composition of the rhizosphere microbiome have been revealed in previous studies (Mukhtar et al., 2018; Wang et al., 2020). Higher relative abundances of arbuscular mycorrhizal fungi (AMF) were observed in treatments that support plant growth (M and WIM) than in the Control soil (Figure 5C). This could be due to the increase in the abundance of AMF that were in symbiosis with cotton, since most of the AMF were within the family Glomeraceae (Supplementary Table S3), members of which have been reported to be commonly associated with cotton (Nehl and McGee, 2010). Furthermore, previous studies reported that AMF can alleviate salt stress and promote growth and yield in cotton (Khaitov and Teshaev, 2015).

Nitrogen is one of the most important elements influencing crop yield (Greenwood, 1982), and nitrogen transformations in soil are primarily driven by soil microbes (Simon and Klotz, 2013; Sun et al., 2015a). The current study revealed that some functional groups involved in nitrogen cycling were significantly impacted by the amelioration practices. Notably, some archaeal and bacterial functional groups catalyzing the same nitrogen cycling processes responded differently to the amelioration practices (Figure 5). The archaeal aerobic ammonia oxidation functional groups increased in WI- and WIM-treated soils, whereas their bacterial counterparts were not significantly affected. This observation was supported by qPCR results from the archaeal and bacterial *amoA* genes, which showed that the abundance of the archaeal *amoA* gene was significantly increased by WI and WIM ameliorations, but bacterial *amoA* gene abundance showed minimal variation across treatments (Supplementary Figure S2). The different responses of oxidizing archaea (AOA) and bacteria (AOB) to agricultural management and their niche separation have been widely reported (Schleper, 2010; Prosser and Nicol, 2012; Stahl and de la Torre, 2012; Segal et al., 2017; Xiang et al., 2017; Sun et al., 2019). In the present study, the higher abundance and greater sensitivity of AOA compared with AOB might indicate their important role in ammonia oxidation in this coastal saline soil.

## Conclusion

In this study, the microbial community composition, diversity, assembly mechanisms and potential functions in costal saline soil under the effects of freezing saline water irrigation and plastic mulching were investigated. Microbial communities in coastal saline soil are composed of a high relative abundance of marine-associated taxa, which were reduced by amelioration practices. Amelioration practices exerted contrasting effects on the richness and vertical distribution among bacterial, archaea and fungal communities. β-null deviation analysis showed that the deterministic process played a dominant role in microbial community assembly in this coastal saline soil and that its effect was decreased in ameliorated soil. These results, combined with the significant correlation between soil microbial communities and soil salinity, indicated that the amelioration practices led to a transition of microbial communities primarily through weakening the strong selectivity of high salinity and forming a microbial community more suitable for agricultural production. These findings advanced our understanding of the mechanisms underlying coastal saline soil amelioration and provided valuable information for the development of potential biofertilizers for use in salt-affected soils.

## Data Availability Statement

The datasets presented in this study can be found in online repositories. The names of the repository/repositories and accession number(s) can be found below: https://www.ebi.ac.uk/ena, PRJEB29290.

## Author Contributions

BL, XL, and RS conceived the project. RS, XW, and YT performed the laboratory experiments and analyzed the data. KG, XF, HS, and XL designed and setup the field experiments. RS and BL wrote the manuscript. All authors discussed the results and approved the final version of the manuscript.

## Conflict of Interest

The authors declare that the research was conducted in the absence of any commercial or financial relationships that could be construed as a potential conflict of interest.

## Publisher’s Note

All claims expressed in this article are solely those of the authors and do not necessarily represent those of their affiliated organizations, or those of the publisher, the editors and the reviewers. Any product that may be evaluated in this article, or claim that may be made by its manufacturer, is not guaranteed or endorsed by the publisher.
