# Supplementary material for: Long-Term Amelioration Practices Reshape the Soil Microbiome in a Coastal Saline Soil and Alter the Richness and Vertical Distribution Differently Among Bacterial, Archaeal, and Fungal Communities
Source: Front Microbiol. 2022 Jan 11;12:768203. doi: 10.3389/fmicb.2021.768203 (PMC8787143; doi:10.3389/fmicb.2021.768203)
Supplement: Supplementary file 1 [file Data_Sheet_1.docx]

Figure S1. Venn diagrams illustrating the distribution of genera of the archaeal, bacterial and fungal communities from the control and three amelioration treatments (WI, M and WIM). The analysis was performed on the top soil layer (0–10 cm) where the strongest effect of amelioration practices on microbial community was observed.


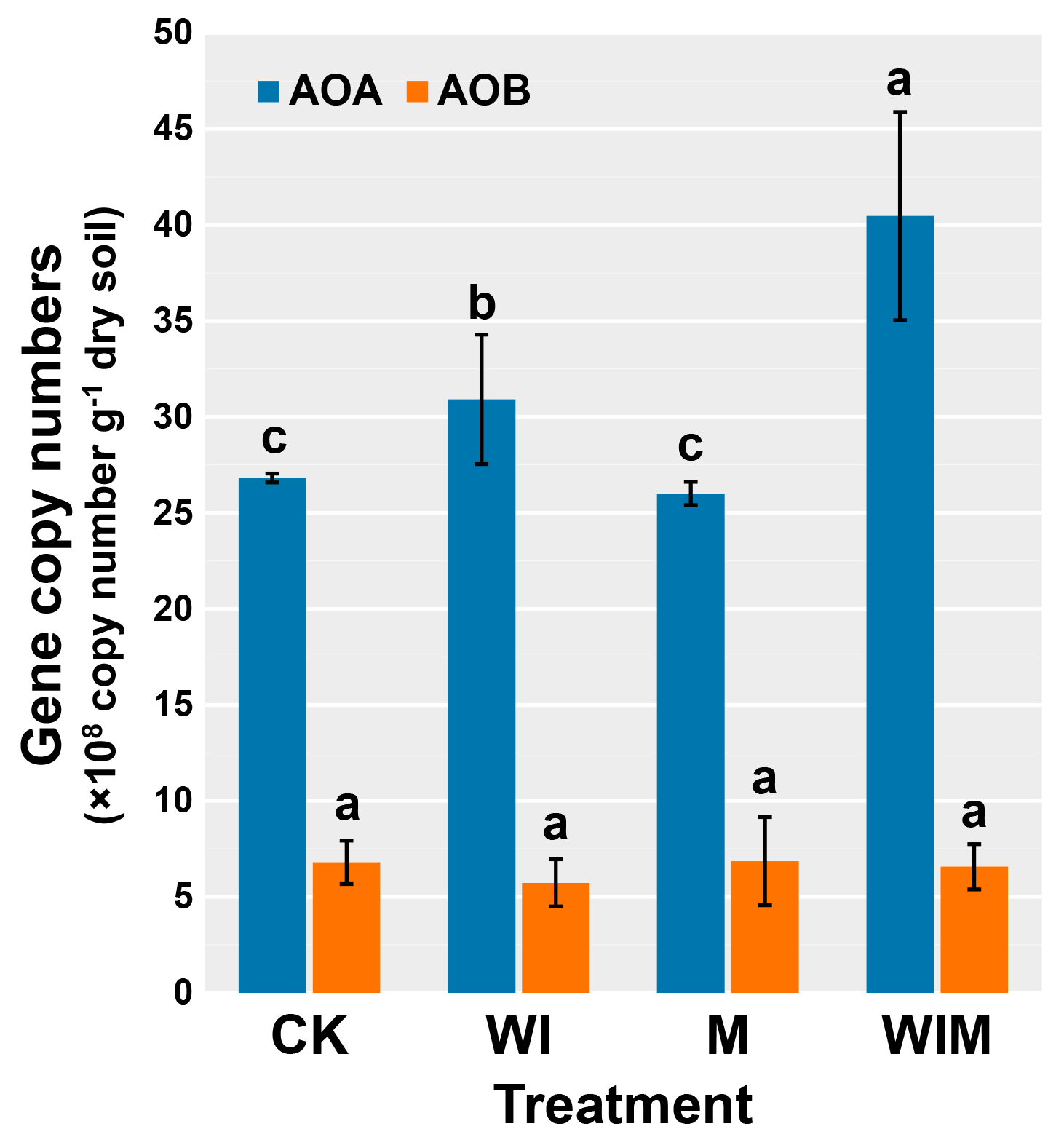


Figure S2. Archaeal (AOA) and bacterial (AOB) *amoA* gene abundance was assessed using qPCR and data are means and standard errors of 3 repeats. Bars with different letters (shown above each) are signiﬁcantly different (*P* <0.05) as revealed by Kruskal-Wallis rank sum test. CK: Control; WI: freezing saline water irrigation; M: plastic mulching; WIM: WI+M.

Table S1. Results of ANOSIM based on Bray–Curtis distance.

| Comparisons among treatments within a soil layer | | | | | | |
| --- | --- | --- | --- | --- | --- | --- |
| Soil layer (cm) | archaea | | Bacteria | | fungi | |
|  | r | *P* | r | *P* | r | *P* |
| 0–10 | 0.4815 | 0.004 | 0.5988 | 0.001 | 0.4105 | 0.004 |
| 10–20 | 0.0679 | 0.277 | 0.3241 | 0.402 | 0.5247 | 0.002 |
| 20–30 | 0.09568 | 0.155 | 0.142 | 0.069 | 0.3117 | 0.028 |
| Comparison among soil layers according to treatment | | | | | | |
| Treatment | Archaea | | Bacteria | | Fungi | |
|  | r | *P* | r | *P* | r | *P* |
| CK | 0.6397 | 0.004 | 0.8601 | 0.005 | 0.3251 | 0.053 |
| WI | 0.6049 | 0.023 | 0.5267 | 0.015 | 0.0370 | 0.349 |
| M | 0.856 | 0.004 | 0.8436 | 0.006 | 0.3309 | 0.055 |
| WIM | 0.8519 | 0.002 | 0.786 | 0.004 | 0.0864 | 0.306 |

ANOSIM: analysis of similarities; CK: control; WI: freezing saline-water irrigation; M: plastic mulching; WIM: WI+M

Table S2. Bray–Curtis distance of soil microbial communities in surface soil according to treatments.

|  | Archaea | | | Bacteria | | | Fungi | | |
| --- | --- | --- | --- | --- | --- | --- | --- | --- | --- |
|  | CK | WI | M | CK | WI | M | CK | WI | M |
| WI | 0.643 |  |  | 0.707 |  |  | 0.679 |  |  |
| M | 0.688 | 0.608 |  | 0.749 | 0.698 |  | 0.686 | 0.728 |  |
| WIM | 0.715 | 0.611 | 0.434 | 0.781 | 0.719 | 0.663 | 0.745 | 0.729 | 0.607 |

CK: Control; WI: freezing saline-water irrigation; M: plastic mulching; WIM: WI+M.
